# Worldwide research tendencies on natural orifice transluminal endoscopic surgery: a bibliometric analysis

**DOI:** 10.3389/fsurg.2026.1567215

**Published:** 2026-02-19

**Authors:** Ji-Xuan Zou, Yan Geng

**Affiliations:** 1 Department of Graduate Student, Guilin Medical University, Guilin, China; 2 Department of Gastroenterology, 923 Hospital of Chinese People’s Liberation Army, Nanning, China

**Keywords:** bibliometric, cholecystectomy, citespace, natural orifice transluminal endoscopic surgery, VOSviewer

## Abstract

**Background:**

Natural orifice transluminal endoscopic surgery, abbreviated as NOTES, a novel surgical technique, involves the insertion of a flexible endoscope into a natural orifice, such as the oral cavity, stomach, vagina, colon, or bladder, to access a specific site for endoscopically assisted surgical procedures. In recent years, endoscopy-mediated NOTES research has witnessed substantial progress.

**Objective:**

This study seeks to offer a bibliometric analysis of the trends and prospects within the NOTES domain.

**Methods:**

On January 3, 2026, this study utilized the Web of Science Core Collection (WOSCC) to retrieve literature related to NOTES. The R package “Bibliometrix” was employed to provide fundamental bibliometric data. VOSviewer was instrumental in conducting keyword analysis, authors analysis, and co-occurrence analysis. Meanwhile, CiteSpace was employed for the analysis of reference bursts and keyword bursts. Moreover, we obtained clinical trial data published within the study period via the PubMed database, aiming to assess clinical advancements in this domain.

**Conclusion:**

This study identified a total of 2,196 publications spanning a 28-year period from 1998 to 2025. The United States led in the number of articles published, with *Surgical Endoscopy And Other Interventional Techniques* being the most prolific journal, and Harvard University emerging as the most productive institution. The keyword “cholecystectomy” has gained considerable popularity in recent years. Clinical trials in this field have focused on the advantages of NOTES over traditional laparoscopic surgery, as well as the surgical efficacy of VNOTES in the treatment of benign gynecological diseases. Through bibliometric analysis, this paper delineates a foundational intellectual framework, suggesting that NOTES is entering a novel phase of development and is poised to retain its academic significance in forthcoming research endeavors.

## 1 Introduction

NOTES is an avant-garde technique wherein a flexible endoscope is navigated through the body's natural orifices, such as the oral cavity, stomach, vagina, colon, and bladder, to reach the target area for endoscopically assisted procedures (1). This approach encompasses the resection of various organs, including the gallbladder, liver, appendix, ovaries, and uterus (2). In recent years, endoscopy-based NOTES has seen rapid evolution, demonstrating significant potential in the management of digestive, urological, and gynecological disorders. As a minimally invasive surgical technique, NOTES has become an integral part of endoscopic therapy, characterized by reduced trauma and scarring (3). By entering the body through a natural lumen, NOTES minimizes damage to external tissues, leading to shorter recovery times, decreased postoperative pain (4), and the absence of visible scarring, which can positively influence patient psychology and cosmesis. With the ongoing advancement of NOTES, an array of new methods have been proposed and successfully implemented clinically, including transgastric appendectomy (5), transgastric cholecystectomy (6), transvaginal appendectomy (7), transvaginal cholecystectomy (8), peroral endoscopic myotomy (POEM) (9), transvaginal hysterectomy (10), transanal total mesorectal excision (trans-TME) (11), transvaginal nephrectomy (12),etc. We conducted a bibliometric analysis of articles published between 1998 and 2025. Bibliometric methodology involves the quantitative and qualitative analysis of specific topics and articles by researchers to evaluate academic potential and development prospects. The objective of this paper is to provide a bibliometric analysis of the trends and future directions of NOTES.

Bibliometrics involves the analysis of published scholarly materials. By employing statistical techniques, it aims to uncover and depict the interrelationships among various academic outputs (13). Presently, bibliometrics is broadly used to analyze publication characteristics in specific academic fields, such as countries, institutions, journals, authors, papers, citations, and keywords. Bibliometrics often uses specific tools to create graphs that visualize cooperation between authors, institutions, and countries (14).

## 2 Methods

### 2.1 Data source and search strategy

**Figure 1:**
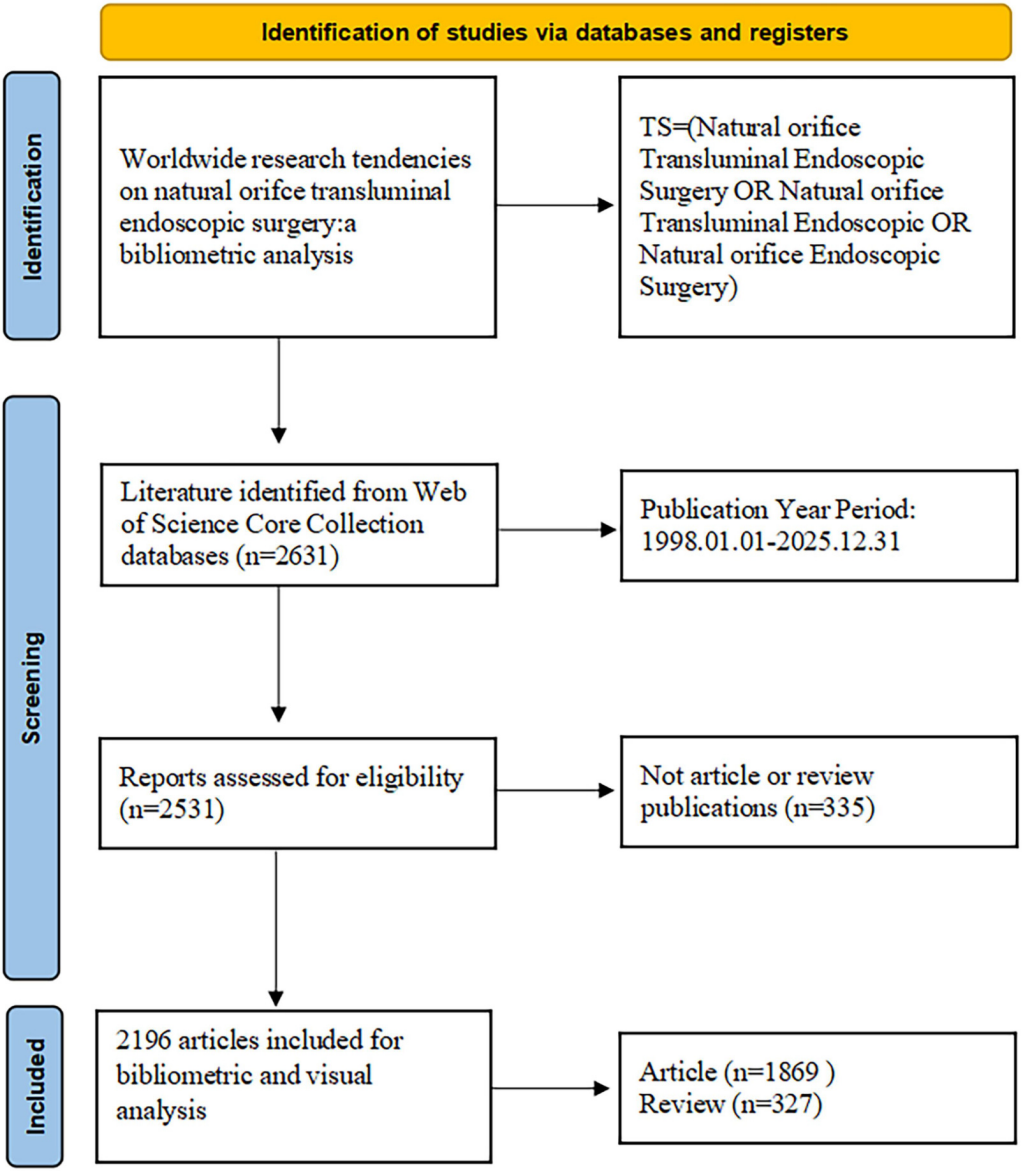
Literature screening flowchart.

Web of Science (WOS) is one of the world's most comprehensive academic databases, with more than 12,000 journals and citation records (15). In this article, the Web of Science Core Collection (WOSCC) was selected as the primary data source for this bibliometric study. Data were retrieved through Guilin Medical University's institutional subscription on January 3, 2026. The search strategy was established in the following manner: TS = (Natural Orifice Transluminal Endoscopic Surgery OR Natural Orifice Transluminal Endoscopy OR Natural Orifice Endoscopic Surgery) AND DOP = (1998-01-01/2025-12-31) AND DT = (Article OR Review) (Note: TS = Topics; DOP = Publication date; DT = Document type). Two independent assessors conducted eligibility screening of all retrieved records by reviewing titles, abstracts, and full-text manuscripts. Discrepancies between reviewers were addressed either through collegial consensus or arbitration by a third independent reviewer. The entire screening workflow is summarized in a PRISMA-compliant flowchart (Figure 1). The remaining publications were archived in plain text format, and their complete bibliographic references were exported as integral datasets. After removing irrelevant records, we identified a total of 2,196 eligible publications. Additionally, given the absence of relevant clinical trial data in WoSCC, we extracted such information from the PubMed database. [“Natural Orifice Transluminal Endoscopic Surgery”[Title/Abstract] OR “Natural Orifice Transluminal Endoscopy”[Title/Abstract] OR “Natural Orifice Endoscopic Surgery"[Title/Abstract]] AND ([“1998/01/01”[Date—Publication]: “2025/12/31”[Date—Publication]]) with the filter: Clinical Trial. Clinical trial outcomes were saved in the standard PubMed output format (14).

### 2.2 Related tools

The bibliometric analysis of this study used three tools: Bibliometrix R package, CiteSpace, and VOSviewer.

Bibliometrix is an analytical visualization tool based on R. The tool collects and analyzes data on the number of publications and citations from countries, institutions, journals, and authors (13).

VOSviewer is a visualization software based on the embedded clustering algorithm (16) for bibliometric analysis in specific research areas. In this study, we used co-authorship analysis to unveil the collaborative efforts among authors and constructed a network diagram to illustrate the connections between different keywords. Moreover, it enhanced the analysis by incorporating a time-overlaying function to depict the network across a temporal range.

CiteSpace is a software utilized for visualization and analysis (17). In this study, CiteSpace was applied to identify references and keywords with strong citations over a certain period of time.

## 3 Results

### 3.1 Annual publication output

**Figure 2:**
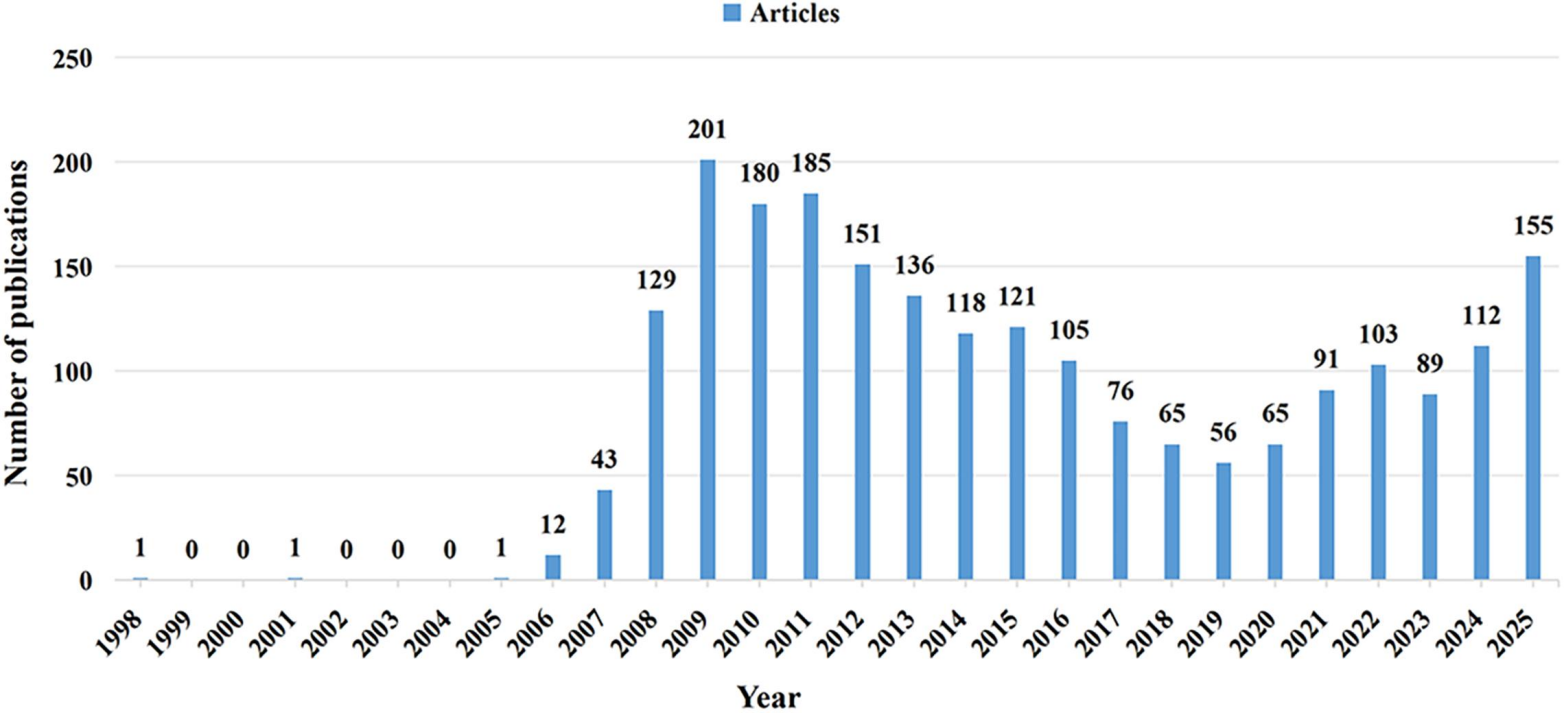
Annual number of publications.

Pursuant to the search protocol, between January 1, 1998, and December 31, 2025, a cumulative total of 2,196 publications pertaining to NOTES were identified, comprising 1,869 original articles and 327 review articles. Analyzing the annual growth rate of these publications, the entire period can be delineated into four distinct phases: Phase I (1998–2005), Phase II (2006–2011), Phase III (2012–2018), and Phase IV (2019–2025). As depicted in Figure 2, Phase I saw a nascent publication count of 3, marking the inception of NOTES research. In 2006, American scholars first formally proposed the concept of Natural Orifice Transluminal Endoscopic Surgery (NOTES) and developed a corresponding white paper (18). Accordingly, the number of publications experienced a substantial surge in the second phase, with an annual average of approximately 125 papers published. This period represented the heyday of NOTES research, among which the year 2009 recorded the largest volume of NOTES-related publications, totaling 201 articles. After 2012, due to the inherent technical complexity of NOTES itself and the rapid iterative advancement of laparoscopic robotic surgery during the same period, the number of NOTES-related publications underwent a gradual decline in the third phase, with an annual average of roughly 110 papers. In recent years, the advent of state-of-the-art flexible endoscopes and robotic technologies (19) has addressed the long-standing issue of insufficient operative space encountered in the early stages, fueling a renewed upward trend in the number of NOTES-related publications in the fourth phase.

### 3.2 Contribution of countries and institutions

**Figure 3:**
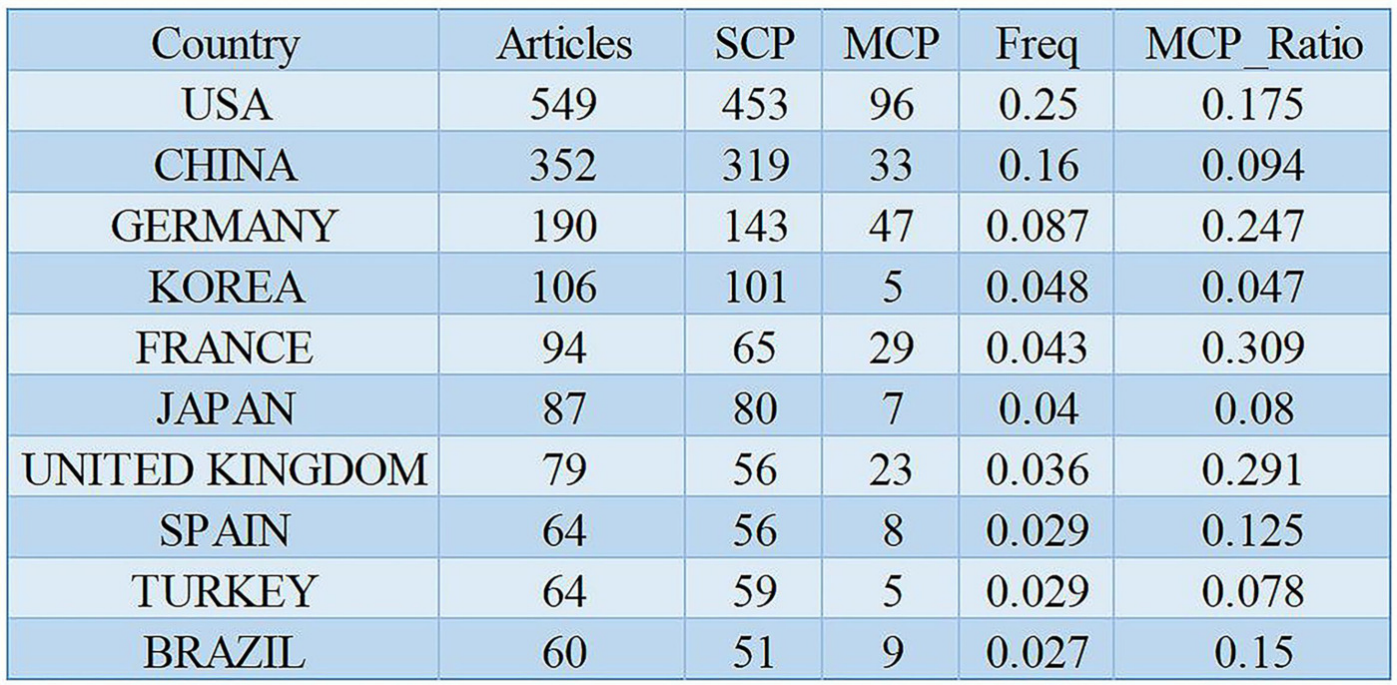
The top 10 countries with the highest publications.

Upon analyzing the distribution of publications by country in the field of NOTES, this paper identified a total of 58 nations contributing to the literature. As illustrated in Figure 3, among these, the United States leads with the highest number of publications (*n* = 549), accounting for 25% of the total. China ranks second with 352 publications (16%), followed by Germany with 190 publications (8.6%). Together, publications from China and the United States constitute nearly half of the total (41%). Multiple country publications (MCP) mirror the joint endeavors of scholars hailing from diverse countries. Although the USA has the highest number of MCP (*n* = 96), the ratio of MCP to total articles is only 17.5%.

A total of 1,706 institutions have conducted research related to NOTES. The top 10 institutions are listed in Table 1. Among these leading institutions, seven are based in the United States, two in Spain, and one in Germany, indicating a substantial commitment to NOTES research by American research institutions. Notably, Harvard University ranks first with the highest output of 80 publications.

**Table 1:** The top 10 institutions with the highest publications.

Rank	Affiliation	Articles
1	Harvard University	80
2	Harvard Medical School	63
3	Massachusetts General Hospital	61
4	Hospital Clinic De Barcelona	58
5	Technical University Of Munich	56
6	University Of Texas System	51
7	University Of Barcelona	48
8	Case Western Reserve University	47
9	University System Of Ohio	46
10	University Of California San Diego	45

These findings indicate that American scholars are the leading contributors to the field of NOTES. This preeminence can be attributed to two core factors: first, the concept of NOTES was originally proposed by American scholars (18); second, prestigious American medical institutions—including Harvard Medical School and Massachusetts General Hospital—launched clinical pilot programs of NOTES at an early stage. These initiatives have enabled the accumulation of extensive surgical data, which in turn has provided pivotal clinical evidence for subsequent scholarly publications.

### 3.3 Analysis of article yield and impact of journals

A total of 462 journals have published the 2,196 articles retrieved in this study. Table 2 presents the top 10 journals, ranked by the total number of articles published. Among these, *Surgical Endoscopy and Other Interventional Techniques*, a journal from the United States, exerts the most significant influence, leading with a total output of 275 articles. The Impact Factor (IF) and quartile rankings are sourced from the Journal Citation Reports (20). Among the top ten journals, five are placed in Q1, indicating a robust academic influence of the aforementioned journals on the field of NOTES research.

**Table 2:** The top 10 journals with the most publications on NOTES.

Rank	Journals	Articles	IF	JCR-c
1	Surgical Endoscopy And Other Interventional Techniques	275	2.4	Q2
2	Gastrointestinal Endoscopy	90	6.7	Q1
3	Surgical Innovation	76	1.2	Q3
4	Endoscopy	71	11.5	Q1
5	Journal Of Laparoendoscopic & Advanced Surgical Techniques	68	1.1	Q3
6	Journal Of Minimally Invasive Gynecology	42	3.5	Q1
7	Minimally Invasive Therapy & Allied Technologies	37	2.0	Q2
8	Journal Of Endourology	35	2.9	Q1
9	Surgical Laparoscopy Endoscopy & Percutaneous Techniques	35	1.1	Q3
10	World Journal Of Gastroenterology	33	4.3	Q1

### 3.4 Contribution of authors

A total of 7,056 authors have contributed to research related to NOTES. Table 3 enumerates the 10 authors with the most extensive publications. The most prolific author is Marescaux, Jacques, who has published a total of 41 articles, with an H-index of 43. In early 2007, Marescaux and his team performed the first transvaginal cholecystectomy in a female using endoscopy. Baekelandt, Jan ranks second in total publication output, with 40 articles published and an H-index of 20. Lin, Yonghong, who ranks third in total publication output, has published 35 articles and has an H-index of 13.

**Table 3:** The 10 authors with the most publications in the field of NOTES.

Rank	Authors	Articles	H-index	Country	Institution
1	Marescaux, Jacques	41	43	France	Universite de Strasbourg
2	Baekelandt, Jan	40	20	Belgium	KU Leuven
3	Lin, Yonghong	35	13	China	University of Electronic Science
4	HE,Li	32	12	China	University of Electronic Science
5	Feussner, Hubertus	28	40	Germany	Technical University of Munich
6	Meining, Alexancler	26	39	Germany	University of Wurzburg
7	Horgan, Santiago	25	42	Usa	Technical University of Munich
8	Correia-Pinto, Jorge	24	31	Portugal	universidade do minho
9	Perretta, Silvana	24	30	France	Universite de Strasbourg
10	Bernard, Dallemagne	23	36	France	Universite de Strasbourg

**Figure 4:**
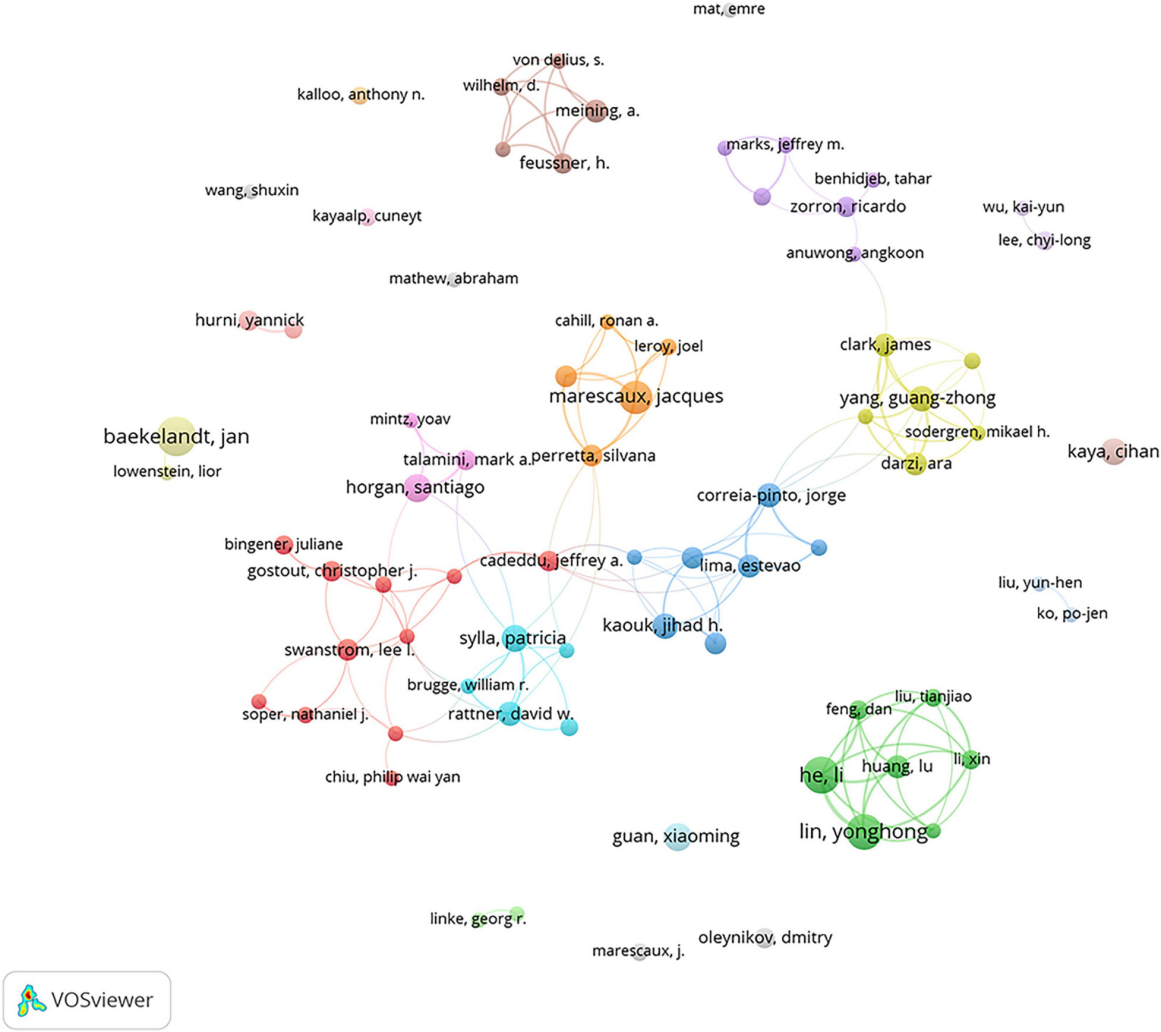
Co-authorship network of authors.

In Figure 4, a co-authorship network analysis of researchers is depicted, unveiling the collaborative ties between them. The larger the node, the greater the number of articles published by the author, while the color indicates author clusters with varying degrees of collaborative strength. A minimum publication threshold of 10 papers per author was set, which resulted in the inclusion of 74 authors; these authors were further categorized into 22 distinct clusters. These clusters are dispersed and do not form a single large community, indicating limited collaboration between different clusters.

**Figure 5:**
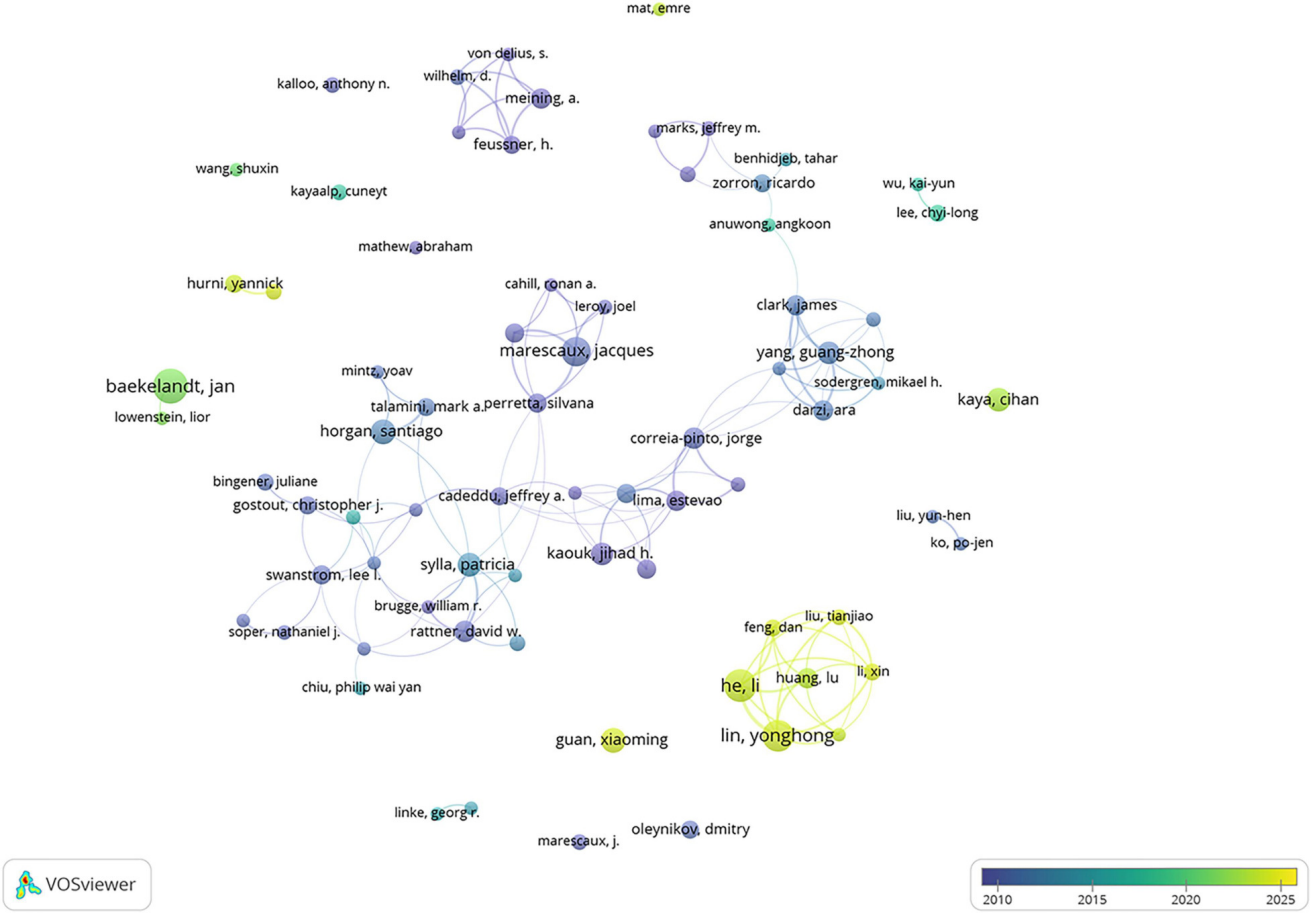
Co-authorship time-overlapping network of authors.

In Figure 5, the temporal overlap graph visualizes the co-authorship network analysis among 74 researchers. Observations indicate that in recent years, researchers Huang Lu, He Li, and Lin Yonghong have been actively contributing to the field of NOTES research.

## 4 Research hotspots

### 4.1 Most cited publications

Highly cited works in a particular field illustrate the significance of the research. Table 4 details the 10 most cited works. As indicated in the list, these were published between 2001 and 2018, with all the articles garnering over 300 citations each. The most cited article was “*ASGE/SAGES working group on natural orifice translumenal endoscopic surgery—October 2005*” (18), published in 2006. The second most cited publication was entitled “*Surgery without scars: report of transluminal cholecystectomy in a human being*” (21), published in 2007.

**Table 4:** Top 10 most cited publications in the field of NOTES.

Rank	Paper	First author, year, journal	Total citations	TC per year
1	ASGE/SAGES working group on natural orifice translumenal endoscopic surgery—October 2005	Rattner d, 2006, Surg Endosc	644	30.67
2	Surgery without scars—Report of transluminal cholecystectomy in a human being	Marescaux j, 2007, Arch Surg-Chicago	628	31.40
3	NOTES transanal rectal cancer resection using transanal endoscopic microsurgery and laparoscopic assistance	Sylla p, 2010, Surg Endosc	591	34.76
4	Endoscopic Transgastric vs. Surgical Necrosectomy for Infected Necrotizing Pancreatitis A Randomized Trial	Bakker Oj, 2012, Jama-j Am Med Assoc	515	34.33
5	Single port access laparoscopic right hemicolectomy	Bucher p, 2008, Int j Colorectal Dis	375	19.74
6	Transoral Endoscopic Thyroidectomy Vestibular Approach: A Series of the First 60 Human Cases	Anuwong a, 2016, World j Surg	366	33.27
7	Minimally invasive and robotic surgery	Mack Mj, 2001, Jama-j Am Med Assoc	358	13.77
8	Safety and Outcomes of the Transoral Endoscopic Thyroidectomy Vestibular Approach	Anuwong a, 2018, Jama Surg	351	39.00
9	Image-Guided Interventions: Technology Review and Clinical Applications	Cleary k, 2010, Annu Rev Biomed Eng	336	19.76
10	Transumbilical Single-Port Surgery: Evolution and Current Status	Canes d, 2008, Eur Urol	329	17.32

### 4.2 Citation burst analysis of references

**Figure 6:**
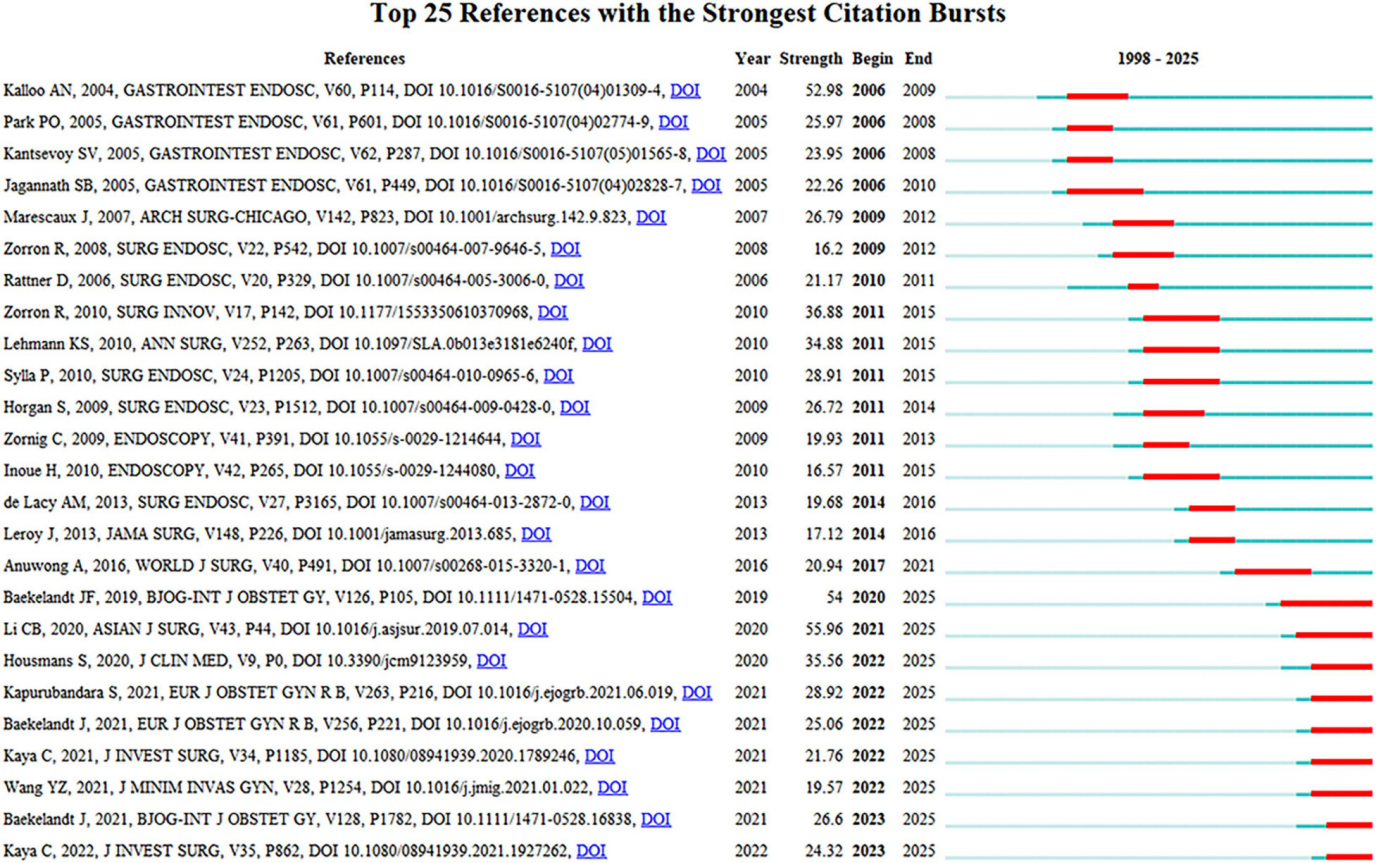
Top 25 references with the strongest citation bursts on NOTES.

In Figure 6, it presents the top 25 references. The blue lines indicate the time of the citation, whereas the red line denotes the period of citation surge. The minimum burst range is set at 1 year. The reference with the highest quantity of citations and the strongest burst strength is the article authored by Li Chun-Boet al., titled “ Transvaginal natural orifice transluminal endoscopic surgery (VNOTES) in gynecologic surgeries: A systematic review” (citation burst = 55.96 from 2020 to 2025). As a systematic review, this article incorporated 33 relevant publications spanning the period from 2012 to 2018. The findings demonstrated that VNOTES has proven to be feasible, safe, and associated with rapid recovery in the management of benign gynecological conditions. Specifically, certain surgical procedures—such as hysterectomy and ovarian cystectomy—can serve as a viable alternative to conventional laparoscopic surgery. Nevertheless, the application of VNOTES in complex surgeries, including those for malignant tumors and endometriosis, remains in the exploratory stage (22). The reference with the second-highest citation count is the article by Kalloo et al., titled “Flexible transgastric peritoneoscopy: a novel approach to diagnostic and therapeutic interventions in the peritoneal cavity” (23) (citation burst = 52.98), in which Kalloo and team members performed the inaugural flexible transgastric peritoneoscopy.

### 4.3 Analysis of keywords

In this study, a total of 2,344 keywords were retrieved. Table 5 displays the top 20 keywords. The top 5 keywords, in order of frequency, are “cholecystectomy”, ”surgery”, “porcine model”, “survival”, and “feasibility”, highlighting that cholecystectomy, the feasibility of NOTES are focal points in NOTES research, with extensive animal experiments already conducted. Among the top 20 keywords, “cholecystectomy” and “hysterectomy” represent specific surgical procedures in NOTES, indicating a keen interest among researchers in these techniques. Nowadays, an increasing number of NOTES-related procedures are being developed, such as transgastric appendectomy (5), transgastric cholecystectomy (6), transvaginal appendectomy, transvaginal cholecystectomy (8), peroral endoscopic myotomy (POEM) (9), transvaginal hysterectomy (10), transanal total mesorectal excision (trans-TME) (11), transvaginal nephrectomy (12), etc. The inclusion of “laparoscopic cholecystectomy”, “Peritoneoscopy”, and “endoscopic surgery” among the top 20 keywords suggests a research emphasis on comparing endoscopic and laparoscopic surgical approaches (24).

**Table 5:** The top 20 most used keywords.

Rank	Words	Occurrences
1	Cholecystectomy	348
2	Surgery	335
3	Porcine model	239
4	Survival	204
5	Feasibility	194
6	Transluminal endoscopic surgery	189
7	Resection	176
8	Peritoneoscopy	145
9	Experience	136
10	Outcomes	106
11	Closure	102
12	Hysterectomy	97
13	Management	97
14	Access	81
15	Laparoscopic cholecystectomy	80
16	Translumenal endoscopic surgery	80
17	Endoscopic surgery	78
18	Notes survival	68
19	Port	68
20	Anastomosis	66

**Figure 7:**
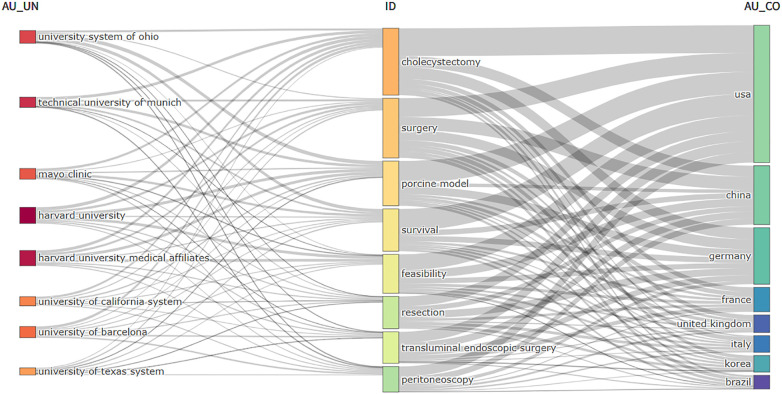
Three-field plot of the keywords (right field: countries; left field: institutions; middle field: keywords;).

Figure 7 additionally delineates the percentage of core themes attributed to each institution and country, highlighting the interconnections and distributions among institutions, keywords, and countries within the domain of NOTES. The majority of institutions and countries have made contributions to the nine themes signified by the keywords. In terms of institutions, Mayo Clinic has shown a greater interest in “Cholecystectomy” and “Survival”, while Harvard University has been more involved with “Feasibility” and “Surgery.” Regarding countries, USA and China have made remarkable contributions to all of these hot topics. Among the nine keywords of “Cholecystectomy, Surgery, Porcine Model, Survival, Feasibility, Resection, Transluminal Endoscopic Surgery, Peritoneoscopy, Experience”, Germany has shown less interest in “Cholecystectomy” and “Peritoneoscopy”, whereas France has paid more attention to “Cholecystectomy” and “Feasibility”.

**Figure 8:**
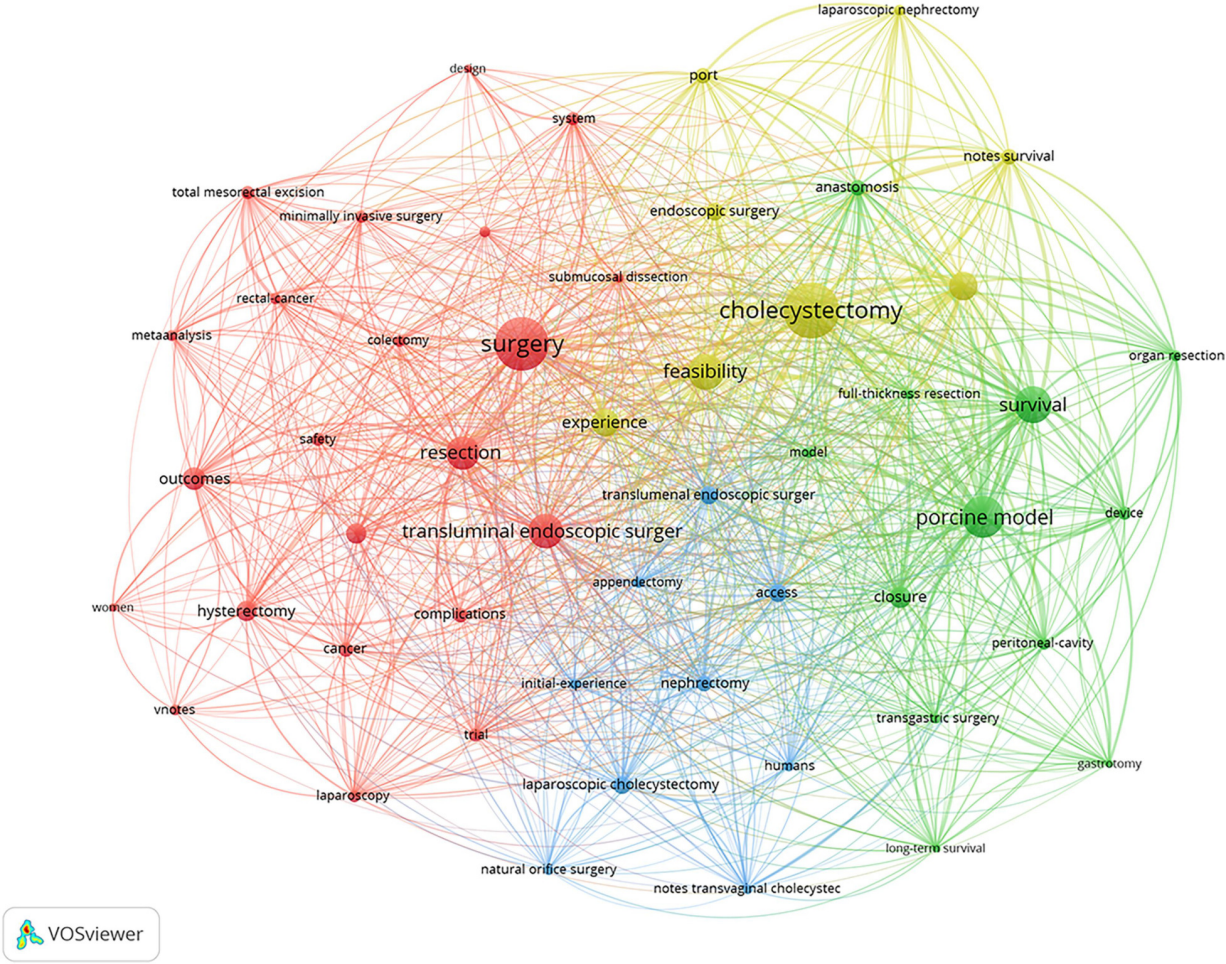
Keywords co-occurrence network.

We set the minimum occurrence threshold for keywords at 33, and a total of 51 keywords were included in the co-occurrence analysis. The network of these keywords is depicted in Figure 8. The size of the nodes represents the frequency of use of the keywords, the color reflects the clusters of keywords, and the distance between nodes indicates the strength of their relationships. Keywords exhibiting tighter associations are categorized within the same cluster. The 51 keywords are divided into 4 clusters. Cluster 1 is red (containing 22 keywords), with the main keywords focusing on NOTES in hysterectomy and colectomy, such as “colectomy”, “hysterectomy”, “minimally invasive surgery”, “resection”, “complications”, ”surgery”. Cluster 2 is blue(containing 9 keywords), with the primary keywords are predominantly focused on appendectomy and nephrectomy, such as “appendectomy”, “nephrectomy,” “design,” “initial-experience”, “system”, and “urology”. Cluster 3 is green(containing 12 keywords), with the main keywords centered on animal experiments, such as “device”, “model”, “porcine model”, “long-term survival”, and “organ resection”. Cluster 4 is yellow (containing 8 keywords), with the main keywords focusing on cholecystectomy, such as “cholecystectomy”, “endoscopic surgery,” “peritoneoscopy”, and “feasibility”.

**Figure 9:**
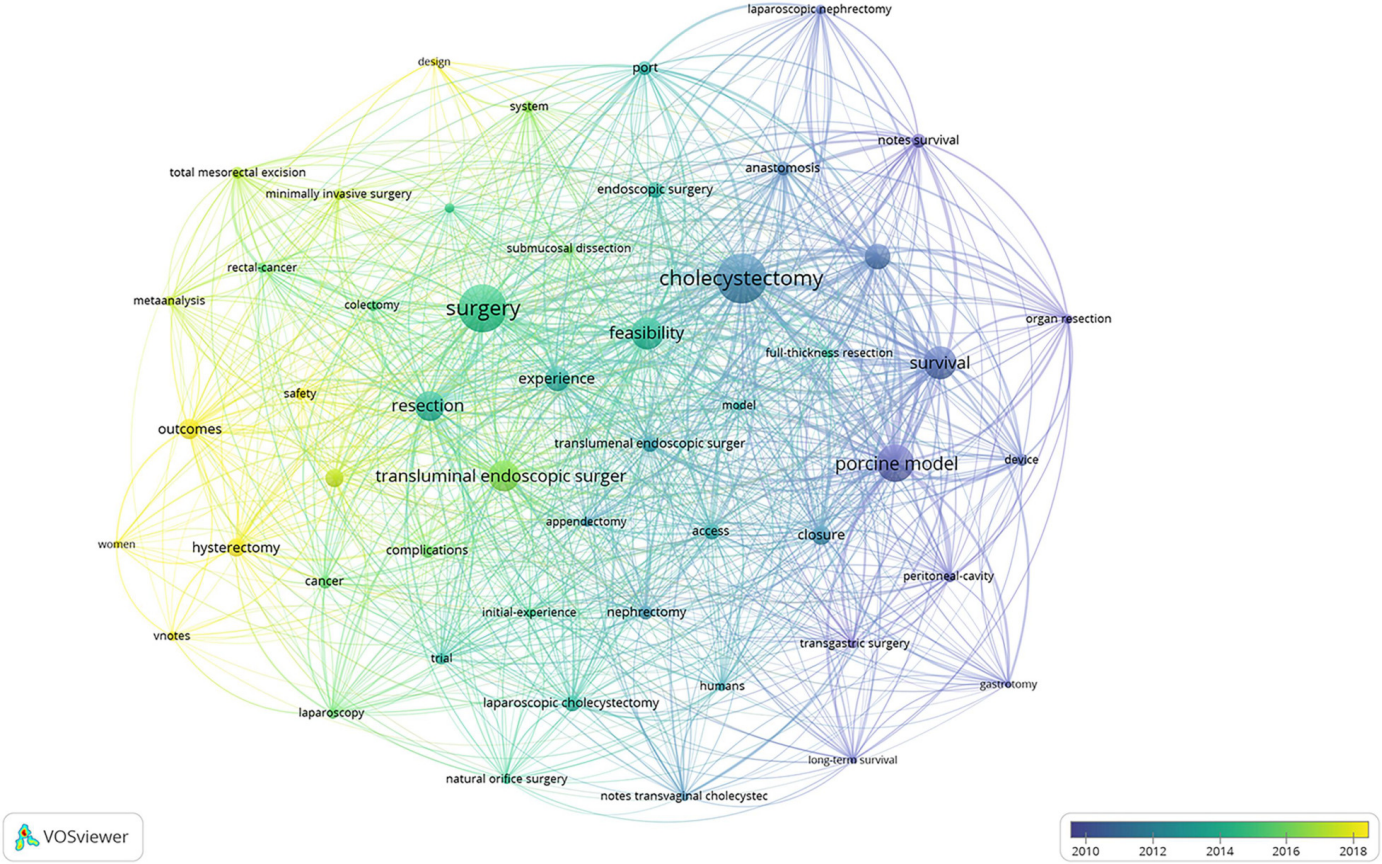
Co-occurrence time-overlapping network of keywords.

Figure 9 illustrates the overlay visualization map analysis network of these co-occurring keywords. The color transition from blue to green, and then to yellow, represents the average active years of these keywords that have attracted researchers’ attention. Early research primarily focused on “peritoneoscopy”, “porcine model”, and “survival”, indicating that early investigators were particularly interested in animal experimentation (25), the distinctions between endoscopic and laparoscopic surgery, and their respective advantages and disadvantages (26, 27). However, topics such as “outcomes”, “minimally invasive surgery”, and “safety” have garnered increasing attention from researchers in recent years, suggesting a growing interest in the outcomes and safety of minimally invasive surgery and NOTES (28). Minimally invasive surgery (MIS) is a core concept of NOTES. Currently, endoscopic minimally invasive therapy has undergone over a decade of vigorous development, and its indications have gradually expanded: from intraluminal to extraluminal, from superficial lesions to deep-seated lesions, and from organic diseases to functional disorders (29).

**Figure 10:**
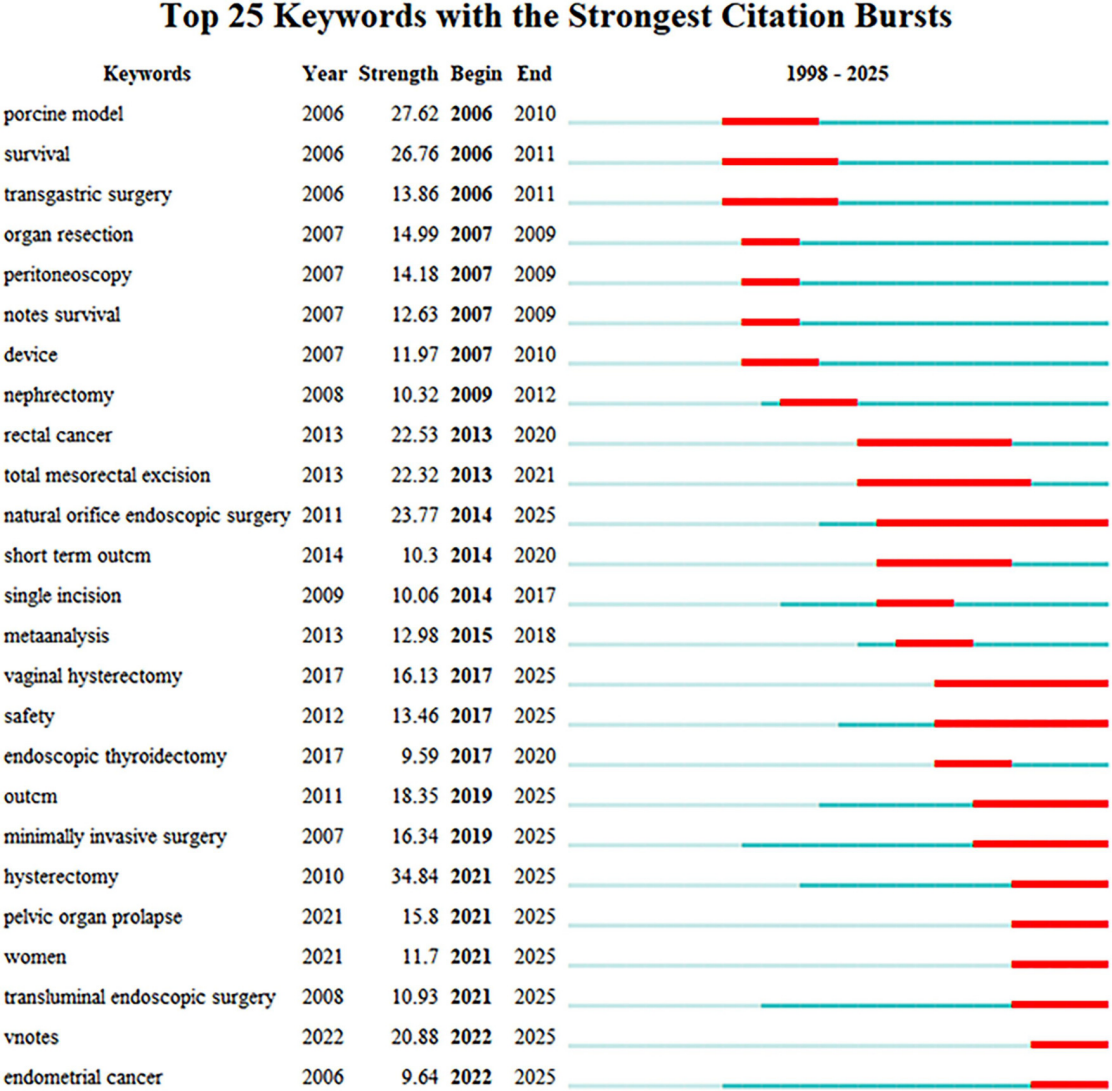
Top 25 keywords with strongest citation bursts.

Figure 10 presents the 25 keywords with the most intense citation bursts. Over time, the keywords “natural orifice endoscopic surgery” (2014–2025, 11 years) and “ transanal total mesorectal” (2013–2021, 8 years) have received the most sustained attention. NOTES plays a pivotal role in transanal total mesorectal excision. Compared with the abdominal approach, the transanal route enables more precise visualization of the anterorectal mesorectal plane in the pelvic floor—an advantage that is particularly pronounced in narrow male pelves (11). Additionally, “hysterectomy” (2021–2025), “pelvic organ prolapse” (2021–2025), “VNOTES” (2022–2025), and “endometrial cancer” (2022–2025) are recent hot topics, suggesting that researchers have begun to extensively investigate the safety and management of complications associated with VNOTES(Vaginal Natural Orifice Transluminal Endoscopic Surgery) in recent years (30,31), foreshadowing that VNOTES represents a popular research topic in recent times and potentially in the future.

### 4.4 Clinical progress analysis

A total of 55 clinical trials were retrieved from the PubMed database. These studies can be broadly categorized into two primary research themes: (1) the advantages of Natural Orifice Transluminal Endoscopic Surgery (NOTES) over conventional laparoscopic surgery; (2) the surgical efficacy of Vaginal Natural Orifice Transluminal Endoscopic Surgery (VNOTES) for the treatment of benign gynecological diseases.

## 5 Discussion

In this article, a bibliometric analysis was conducted to examine the literature related to the field of NOTES published between 1998 and2025. The United States occupies a leading role in this field, having the greatest number of publications. The majority of the top 10 institutions are based in the United States, and the journal *Surgical Endoscopy and Other Interventional Techniques*, also from the United States, leads in terms of publication output with a total of 275 articles, ranking first.

Jacques Marescaux is widely recognized as the most prolific contributor in the field. He and his team pioneered the first transvaginal cholecystectomy using endoscopy in women (21). Additionally, he published the first case of pure transgastric NOTES colorectal surgery (30). Concurrently, he is the principal author of the VNOTES practical guide. His research indicates that hybrid image guidance is a future trend in NOTES, suggesting that robotics and computer science can facilitate the advancement of minimally invasive therapies, converging surgical procedures, advanced endoscopies, and interventional radiology into a new specialty known as hybrid image-guided minimally invasive therapy (31).

One of the issues this study aims to address is identifying the hot topics that researchers widely focus on in the field of NOTES, and the aspects through which researchers can analyze these research hotspots, such as keywords, publications and references. The number of citations a publication receives is a crucial indicator of its impact. Highly cited publications can reflect the core themes of a specific research domain, aiding scientific investigators in pinpointing the research hotspots within that field. Analysis reveals that the 10 most-cited publications related to NOTES concentrate on the themes of safety and feasibility across various natural orifices.

In 2004, Kalloo and his team members conducted the first transgastric peritoneoscopy in a porcine model (23). In October 2005, a scientific task force was established in the United States, to investigate the safety, efficacy, indications, and ethical applications of NOTES in humans, and to disseminate the latest research advancements in NOTES (18). In 2007, Marescaux and colleagues performed the first transvaginal cholecystectomy, concluding that NOTES is feasible and safe (21). David and colleagues summarized the present state and progression of transumbilical endoscopic surgery (32). Angkoon performed the transoral vestibular approach for thyroidectomy, deeming it a safe procedure for patients (28). Numerous studies have shown that NOTES is safe and feasible, and researchers are continuously developing various NOTES procedures through different natural orifices to reduce pain, trauma, and postoperative complications.

Cholecystectomy is the most frequently mentioned keyword in NOTES. Traditional cholecystectomy encompasses open cholecystectomy and laparoscopic cholecystectomy; however, these conventional methods can lead to bile duct injuries and result in large surgical scars that may affect cosmesis (33). In 2007, a seminal article in the journals named *Archives of Surgery* reported the first cholecystectomy performed using NOTES (21), pioneering a new approach to gallbladder removal. Between 1998 and 2024, there were 479 articles with cholecystectomy as the keyword. We consider cholecystectomy to be a primary application direction for NOTES.

Keywords highlight the research hotspots within specific domains. We have additionally performed an extensive analysis of references, summarizing the research hotspots as follows: (1) NOTES procedures, which primarily include transgastric appendectomy (5), transgastric cholecystectomy (6), transvaginal appendectomy (7), transvaginal cholecystectomy (8), peroral endoscopic myotomy (POEM) (9), transvaginal hysterectomy (10), transanal total mesorectal excision (trans-TME) (11), transvaginal nephrectomy (12), etc; (2) The application of robotic technology (34), which can facilitate the execution of complex endoscopic surgeries (35); (3) vNOTES, with hysterectomy being the most common procedure (73%) (36). Vaginal endoscopy can effectively and efficiently perform more complex adnexal surgeries, particularly those related to repair or incontinence procedures (36, 37); (4) The benefits of NOTES for patients, which include scarlessness (3), minimal invasiveness (4), reduced pain (38), and shorter operative times (39); (5) Potential risk factors (40) and complications (36) associated with NOTES.

A review of the PubMed database identified 55 clinical trials focusing on NOTES. An analysis of these studies highlighted key trends and priority areas: (1) The advantages of NOTES over conventional laparoscopic surgery. Specifically, NOTES is comparable to conventional laparoscopic surgery in terms of safety and efficacy, while enabling shorter operative time, reduced postoperative pain, and a lower risk of wound infection (41, 42); it also enhances patients’ postoperative recovery outcomes and decreases the risk of recurrence (43, 44). (2) Surgical Efficacy of VNOTES for the Treatment of Benign Gynecological Diseases. Clinical studies have demonstrated that VNOTES is non-inferior to conventional laparoscopic surgery in terms of adnexectomy or ovarian cyst enucleation (45). VNOTES can be regarded as a more minimally invasive surgical approach, given its ability to reduce postoperative pain, accelerate recovery, and eliminate visible incisions (46, 47); It also exerts no significant adverse effects on female sexual function postoperatively (48). VNOTES has been proven to be a safe, feasible, and minimally invasive therapeutic option (48, 49).

In the near future, research interests may be directed towards several key areas: (1) Exploring a broader range of NOTES procedures; (2) Utilizing robotic technology in the execution of NOTES; (3) Establishing and refining clinical guidelines for NOTES.

Given the annual increase in publications within the field of NOTES, to date, there has only been one bibliometric analysis study published in 2013 (50). Previous bibliometric studies only covered the period from 2006 to 2011, with analytical dimensions focusing on basic aspects such as publication year, article type, study design, journal category, author's specialty, geographical origin, surgical method, and access route. These studies did not explicitly use professional bibliometric tools; instead, they only conducted descriptive analyses through basic statistical methods, failing to involve in-depth analyses such as institutional collaboration, the temporal evolution of keywords, and burst characteristics of highly cited literature. By contrast, this study extends the research period to 1998–2025, fully covering the entire life cycle of NOTES technology from “conceptual germination → rapid development → phased adjustment → recovery and growth,” which enables capturing the long-term evolutionary trends of the technology. Meanwhile, it integrates three mainstream tools: Bibliometrix R package (for basic bibliometric data calculation), VOSviewer (for collaboration network visualization), and CiteSpace (for citation burst analysis). The synergistic use of these tools achieves multi-dimensional analysis encompassing “quantitative statistics + visual presentation + cutting-edge identification,” making the methodology more in line with the norms of modern bibliometric research. However, the study has several limitations (51). Due to the lag in citation impact, some recently published high-quality studies may have been underestimated in their influence and require tracking and updating in future research (14). Despite these limitations, this study will contribute to the academic community's understanding of the development frontiers, trends, and hotspots in the field of NOTES.

## 6 Conclusions

In summary, the field of NOTES is attracting growing interest, with recent research in this area advancing rapidly. Potential future research directions include: (1) Identifying alternative natural orifices beyond the oral cavity, stomach, anus, and vagina for performing NOTES and developing additional procedures that offer benefits to patients; (2) Utilizing robotic technology to assist in NOTES; (3) Establishing and refining clinical guidelines for NOTES. Armed with the analytical results of this study, researchers can conduct more precise and in-depth studies in the field of NOTES and achieve new breakthroughs.

## Data Availability

The original contributions presented in the study are included in the article/Supplementary Material, further inquiries can be directed to the corresponding author.
